# Vector-Borne Viral Diseases

**DOI:** 10.1155/2015/582045

**Published:** 2015-03-24

**Authors:** Penghua Wang, Fengwei Bai, Gong Cheng, Jianfeng Dai, Michael J. Conway

**Affiliations:** ^1^Section of Infectious Diseases, Yale University School of Medicine, New Haven, CT 06512, USA; ^2^Department of Biological Sciences, University of Southern Mississippi, Hattiesburg, MS 39406, USA; ^3^Department of Basic Medical Sciences, School of Medicine, Tsinghua University, Beijing 100084, China; ^4^Institute of Biology and Medical Sciences, Jiangsu Key Laboratory of Infection and Immunity, Soochow University, Suzhou 215123, China; ^5^Central Michigan University College of Medicine, CMED, Mount Pleasant, MI 48859, USA

In this special issue, we have solicited review articles on West Nile virus epidemiology and research articles on dengue diseases.

N. J. Samanta et al. report the occurrence of West Nile virus antibodies in wild birds, horses, and humans serum samples collected between 2010 and 2014 from different areas of Poland. By using multiple ELISA screening, they found West Nile virus positive serum in wild birds, patients, and horses. They suggest that West Nile virus is already present in their climate zone and in their ecosystem.

By using a meta-analysis, H. Zhang et al. study the association between 11 clinical symptoms and the outcome of dengue diseases. They found five symptoms demonstrating an increased risk for severe dengue diseases (SDD), including bleeding, vomiting/nausea, abdominal pain, skin rashes, and hepatomegaly. They concluded that bleeding (hematemesis/melena), vomiting/nausea, abdominal pain, skin rashes, and hepatomegaly may predict the development of SDD in patients with DF.

J. Cime-Castillo et al. report the role of mosquito sialic acid in the life cycle of dengue virus. They identified a putative enzyme involved in sialic acid synthesis and evaluated its function in vitro. Sialic acid interactions were found to be important for dengue virus binding and perhaps transmission.

C. Napoli et al. describe the West Nile virus surveillance system of Italy, which integrates data from human and animal infections.

C. Chancey et al. review the global epidemiology of West Nile virus.

D. Di Sabatino et al. review the main epidemiological findings on WNV occurrence in Europe and the Mediterranean Basin from 2009 to 2013 and discuss potential future spread patterns.

*Penghua  Wang* *Fengwei Bai* *Gong Cheng* *Jianfeng Dai* *Michael J. Conway*

